# Short and Long-Term Voice and Swallowing-Related Quality of Life in Patients Who Underwent Total Laryngectomy and Tracheoesophageal Puncture

**DOI:** 10.7759/cureus.27609

**Published:** 2022-08-02

**Authors:** Hani Z Marzouki, Nada E Al Taylouni, Albaraa Tonkal, Ibrahim Amer, Lamis K Halawani, Manal Khoja, Mazin Merdad

**Affiliations:** 1 Otolaryngology - Head and Neck Surgery, King Abdulaziz University Hospital, Jeddah, SAU; 2 Medicine, King Abdulaziz University Hospital, Jeddah, SAU; 3 Speech Therapy, King Abdulaziz University Hospital, Jeddah, SAU

**Keywords:** quality of life, total laryngectomy, laryngectomy, dysphagia handicap index, voice handicap index

## Abstract

Background

Voice and swallowing disorders can create a profound psychosocial impact on the patient’s quality of life. The focus of this study is to assess the psychosocial disabling effects on patients after total laryngectomy (TLP) using pre-validated surveys called the Voice Handicap Index 10 (VHI) for voice disorders and the Dysphagia Handicap Index (DHI) for swallowing disorders.

Methodology

This is a retrospective cohort study that was done on a total of 21 patients. The study was conducted at King Abdulaziz University Hospital between 2017 and 2022. The Cronbach’s alpha value was used to evaluate internal consistency reliability. The relationship between DHI, VHI, and demographic and clinical variables was analyzed by correlation analysis. A p-value of <0.05 was considered statistically significant, and all the tests were two-sided.

Results

The Cronbach’s alpha coefficient satisfied the internal consistency reliability for VHI and DHI for both total and their subscale scores. For DHI, the total score and emotional subscale obtained an alpha of greater than 0.9, indicating excellent internal consistency, whereas both physical and functional alpha scores indicated good internal consistency (α = 0.888 and α = 0.863, respectively). For VHI, the total score and physical subscale of the VHI had excellent internal consistency (α = 0.957 and α = 0.937, respectively), while the functional and emotional subscales had good internal consistency (α = 0.865 and α = 0.894, respectively). The total DHI scores, as well as the functional subscale scores, were significant (p = 0.033, p = 0.025, respectively) in terms of self-reported dysphagia severity. A moderately severe group (69.00 ± 19.17) had higher mean total scores, whereas severe individuals had higher subscale mean scores according to self-reported dysphagia severity. Self-reported dysphagia severity was as follows: normal (n = 3, 14.3%), mild (n = 4, 19%), moderate (n = 8, 38.1%), and severe (n = 6, 28.6%).

Conclusions

The disability caused by voice and swallowing disorders can be evaluated by VHI and DHI which have been statistically validated as reliable tools to assess the effects of dysphagia and dysphonia on quality of life. Patients after laryngectomy have higher mean DHI and VHI overall and functional subscale scores. However, this study failed to establish any relationship between clinical and demographical characteristics of the patients with DHI and VHI index.

## Introduction

Patients treated with total laryngectomy are at risk of several consequences. Post-laryngectomy, patients experience issues such as obstructed airways, voice difficulties, decreased physical strength, and diminished sensations of smell and taste. However, in the last 25 years, attempts have been undertaken to improve laryngectomy rehabilitation. The commonly reported adverse effects after total laryngectomy are voice-related problems (66-84%) [1,2] and dysphagia (up to 72%) [3-6]. Both of these complications have the potential to negatively impair one’s health-related quality of life. Dysphagia is related to various negative health effects, including poor nutrition, weight loss, and a lower quality of life. Up to 40% of patients have reported severe distress as a result of dysphagia symptoms following total laryngectomy [4]. Voice therapy helps many laryngectomees regain a functional voice and understandable speech; however, some may require further communication aids, such as learning sign language, or the use of an electrolarynx. Alterations in voice function, as well as other bodily changes, persist not only for a short time following laryngectomy but also for the rest of one’s life [2].

To examine patient-reported outcomes, self-reported questionnaires are routinely utilized. They ensure that questions are asked in a consistent manner and may make data collection for postoperative dysphagia and voice difficulties easier. Examining the viewpoints of patients may be beneficial to a more successful recovery. Voice Handicap Index 10 (VHI) is a self-assessment tool designed to examine the quality of life of people experiencing voice disorders and evaluate the patient’s awareness, their self-perception impression, and the impact caused by their voice disorders. The VHI has been validated as well as translated into numerous languages. Until now, few researchers have used the VHI and the Dysphagia Handicap Index (DHI) to measure psychosocial aspects after total laryngectomies. The VHI contains 30 questions segregated into three subscales, namely, functional, physical, and emotional. The functional component of VHI focuses on the impact of voice disorders on daily functions and activities. The physical scale concerns the subjective feelings due to laryngeal discomfort, whereas the emotional scale elicits the impact of voice disorders on the respondent’s psychological and emotional well-being [7]. Each item is scored on a five-point Likert-type scale with scores ranging from 0 to 4, indicating “Never” to “Always.” The overall score ranges from a minimum of 0 to a maximum of 120, where lower scores indicate lesser severity and higher scores imply a greater voice handicap [8] DHI, designed on the VHI model, is also a diagnostic tool to assess the impact on quality of life due to swallowing disorders. Similar to VHI, it includes 25 statements on dysphagia-related aspects of daily life with a five-point rating scale and is subdivided into three domains. The physical component focuses on symptoms, the functional domain elicits nutritional and respiratory consequences, whereas the emotional component focuses on psychosocial consequences [9].

The goal of this study was to validate the VHI and DHI to assess voice and dysphagia handicaps in a group of total laryngectomy patients, as well as to investigate the psychosocial aspects of voice and dysphagia handicapping.

## Materials and methods

Patients and study design

This retrospective cohort study was conducted between 2017 and 2022 at King Abdulaziz University Hospital. We included patients who underwent total laryngectomy and tracheoesophageal puncture. A total of 21 patients were identified and recruited to answer the VHI and DHI surveys which were translated by two independent translators. Signed consent was obtained from each participant to be enrolled in the study. The data were answered and entered on a Google Forms sheet and were collected in the clinic through interpersonal interviews with the patients.

Statistical analysis

The data were entered into Microsoft Excel, which cleaned it all up for analysis. Variables were then imported into SPSS version 26 (IBM Corp., Armonk, NY, USA) for statistical analysis. Kolmogorov-Smirnov test was applied to assess the type of data distribution. The Cronbach’s alpha values of 0.7-0.8, 0.8-0.9, and >0.9 were considered acceptable, good, and excellent internal consistency, respectively [10]. The relationship between the DHI, VHI, and baseline and clinical variables was analyzed using correlation (Pearson). The independent-sample t-test and analysis of variance (ANOVA) were followed by a post hoc test. A p-value of <0.05 was considered statistically significant, and all tests were two-sided.

## Results

In this study, the DHI and VHI were administered to 21 patients (20 males and one female). The mean age was 58.45 ± 12.36 years, ranging from 34 to 85 years. The education level of the patients was classified as follows: none (19%), primary (14.3%), secondary (23.8%), bachelor and equivalent (28.6%), and masters (4.8%). Of the total patients, 47.6% were exposed to radiation before, 38.1% received postoperative radiotherapy, and 42.9% received multiple sessions of radiotherapy.

Reliability analysis

The internal consistency reliability of the DHI and VHI was evaluated using Cronbach’s alpha. The items contained within the subscales with a high item-total correlation contributed to the total reliability. In the DHI, the total score and emotional subscale obtained an alpha of greater than 0.9, indicating excellent internal consistency. Both physical and functional alpha scores indicated good internal consistency (α = 0.888 and α = 0.863, respectively). The total score and physical subscale of the VHI had excellent internal consistency (α = 0.957 and α = 0.937, respectively), while the functional and emotional subscales had good internal consistency (α = 0.865 and α = 0.894, respectively) (Table 1).

**Table 1:** Reliability analysis of the total and subscale scores of DHI of patients. DHI: Dysphagia Handicap Index; VHI: Voice Handicap Index

	DHI		VHI	
	Number of items	Alpha	Number of items	Alpha
Subscale				
Physical	9	0.888	9	0.937
Functional	9	0.863	10	0.865
Emotional	8	0.936	10	0.894
Total	25	0.958	29	0.957

Correlation analysis of the DHI

The DHI questions are graded on a four-point Likert scale (0 = never, 1 = almost never, 2 = sometimes, and 3 = always). Total scores and subscale scores can range as follows: DHI overall (n = 25, 0-100), physical domain (n = 9, 0-36), functional domain (n = 9, 0-36), and emotional domain (n = 7, 0-28). Self-reported severity of dysphagia scores can range as follows: 1 indicates normal, 2 and 3 suggest mild, 4 and 5 indicate moderate, and 6 and 7 indicate severe dysphagia. The following is the distribution of patients based on their self-reported dysphagia severity: normal (n = 3, 14.3%), mild (n = 4, 19%), moderate (n = 8, 38.1%), and severe (n = 6, 28.6%). The total scores of DHI and subscales were calculated. The mean overall score was 44.00 ± 29.98. The lowest and highest mean scores were reported for the emotional (12.67 ± 10.13) and functional (16.57 ± 10.00) subscales, respectively. The correlation coefficient between the total scores and subscales, physical, functional, and emotional, were found to be high (r = 0.936-0.958; p = <0.001) and between the subdomains. (functional and physical: r = 0.851; emotional and physical: r = 0.791; and functional and emotional: r = 0.865: r range = 0.791-0.865; p < 0.001) (Table 2).

**Table 2:** Pearson correlation matrix for total and subscale scores for the DHI. DHI: Dysphagia Handicap Index; SD: standard deviation; *: p-value of <0.001

	Mean ± SD	Total	Physical	Functional	Emotional
Total	44.00 ± 29.98	1			
Physical	14.76 ± 10.59	0.936*	1		
Functional	16.57 ± 10.00	0.958*	0.851*	1	
Emotional	12.67 ± 10.13	0.937*	0.791*	0.865*	1

Relationship of the DHI with demographic and clinical factors

Based on the Pearson correlation analysis, age (r = -0.05, p = 0.831) and education (r = 0.036, p = 0.885) levels were not significantly correlated with the total scores, as well as none of the subscales of the DHI. Regarding occupation, non-working patients had higher mean scores in the total and subscale scores of the DHI than retired and working patients, despite the lack of statistical significance. The total scores of the DHI were significantly different among different self-reported severity by the patient (p = 0.033), and severity scores were different among functional subscale scores (r = 0.025). Patients in the moderately severe group (69.00 ± 19.17) had higher mean total scores, while severe patients had higher subscale mean scores. Most patients belonged to the moderate category, followed by severe, mild, and normal. There was no statistically significant difference between self-reported severity classification and the physical (p = 0.057) and emotional (p = 0.1) subscale scores of the DHI. When comparing other clinical characteristics of the patients, those who had been exposed to radiation and had previously undergone radiation therapy had higher mean total and subscale scores compared to other patients. Patients who underwent multiple radiation therapy sessions had lower total DHI scores compared to others. However, there was no statistical significance between the mean scores of the DHI and the clinical factors of the patients (Table 3).

**Table 3:** Pearson correlation matrix for total and subscale scores for the VHI. VHI: Voice Handicap Index; SD: standard deviation; *: p-value of <0.001

	Mean ± SD	Total	Physical	Functional	Emotional
Total	65.38 ± 34.29	1			
Physical	20.81 ± 12.35	0.917*	1		
Functional	23.86 ± 11.37	0.891*	0.71*	1	
Emotional	20.71 ± 13.62	0.942*	0.81*	0.764*	1

Correlation analysis of the VHI

The VHI items are graded on a four-point Likert scale (0 = never, 1 = almost never, 2 = sometimes, and 3 = always). Total scores and subscale scores can range as follows: VHI total (29 items, 0-116), physical (9 items, 0-36), functional (10 items, 0-40), and emotional (10 items, 0-40). The VHI mean scores of both total and subscales were calculated. Lower scores indicate a better quality of life. The mean total score was 65.38 ± 34.29. The lowest and highest scores were reported for the emotional (20.71 ± 13.62) and functional (23.86 ± 11.37) subscales, respectively. The correlation coefficient between the total scores and their subscales was high, as well as for the subscales (r = 0.71 to r = 0.942; p < 0.001) (Table 4).

**Table 4:** Univariate analysis of the relationship between total and subscale scores of DHI with the demographic and clinical factors of the patients (n = 21). DHI: Dysphagia Handicap Index; *: p-value of <0.05

	Physical		Functional		Emotional		Total score	
	Correlation coefficient (r)	P-value	Correlation coefficient (r)	P-value	Correlation coefficient (r)	P-value	Correlation coefficient (r)	P-value
Age	0.028	0.905	-0.042	0.856	-0.129	0.577	-0.05	0.831
Education level score	0.143	0.558	-0.147	0.548	0.101	0.681	0.036	0.885
Gender								
Male	14.10 ± 28.00		15.9 ± 9.76		11.90 ± 9.74		41.90 ± 28.04	
Female	28		30		28		86	
Occupation								
None	20.00 ± 9.38		22.75 ± 6.92		15.50 ± 8.99		58.25 ± 23.29	
Retired	11.14 ± 11.42		13.71 ± 10.55		12.29 ± 11.63		37.14 ± 31.58	
Employed/working	12.00 ± 9.88		11.67 ± 9.91		9.33 ± 10.41		33.00 ± 29.47	
P-value	0.21		0.072		0.55		0.209	
Severity								
Normal	6.00 ± 2.00		8.00 ± 5.29		7.33 ± 5.03		26.00 ± 21.79	
Mild	9.50 ± 6.81		10.50 ± 9.00		6.00 ± 7.12		42.75 ± 30.89	
Moderate	14.25 ± 11.63		16.25 ± 10.66		12.25 ± 9.29		69.00 ± 19.17	
Severe	23.33 ± 8.55		25.33 ± 3.93		20.33 ± 11.20		44.00 ± 28.98	
P-value	0.057		0.025^a^		0.1		0.033*	
Other clinical characteristics								
Exposed to radiation								
Yes	17.60 ± 11.84		19.00 ± 9.67		15.40 ± 9.94		52.00 ± 30.75	
No	12.18 ± 9.10		14.36 ± 10.23		10.18 ± 10.10		36.73 ± 27.00	
P-value	0.252		0.301		0.248		0.237	
Radiation therapy								
Yes	18.60 ± 10.54		19.60 ± 10.27		14.00 ± 11.07		52.20 ± 29.99	
No	11.27 ± 9.80		13.81 ± 9.35		11.45 ± 9.55		36.54 ± 27.23	
P value	0.115		0.193		0.578		0.225	
Multiple sessions of radiation therapy								
Yes	15.33 ± 9.80		16.44 ± 10.28		9.11 ± 8.84		40.89 ± 27.64	
No	14.33 ± 11.56		16.67 ± 10.25		15.33 ± 10.56		46.33 ± 30.95	
P-value	0.961		0.837		0.169		0.681	

Relationship of the VHI with demographic and clinical factors

The total VHI scores were not significantly correlated with age (r = 104, p = 0.653) and level of education scores (r = -0.338, p = 0.157). None of the VHI subscales were significantly correlated with the age and education level scores of the patients, according to the Pearson correlation analysis results. Despite the absence of statistical significance, non-working patients had higher mean scores on total VHI subscales than retired and working patients. When other clinical characteristics of the patients were evaluated, those who had been exposed to radiation had higher mean total and subscale scores. Total scores of patients who underwent multiple sessions of radiotherapy had lower total scores of the VHI compared to others. However, there was no statistical significance observed between the mean scores of the VHI and the clinical factors of the patients (Table 5).

**Table 5:** Univariate analysis of the relationship between total and subscale scores of the VHI with the demographic and clinical factors of the patients (n = 21). DHI: Voice Handicap Index

	Physical		Functional		Emotional		Total score	
	Correlation coefficient	P-value	Correlation coefficient	P-value	Correlation coefficient	P-value	Correlation coefficient	P-value
Age	0.157	0.495	0.028	0.906	0.097	0.676	0.104	0.653
Education level	-0.195	0.424	-0.307	0.201	-0.43	0.066	-0.338	0.157
Gender								
Male	20.20 ± 12.34		23.05 ± 11.02		19.90 ± 13.44		63.15 ± 33.58	
Female	33		40		37		110	
Occupation								
None	23.75 ± 13.31		29.38 ± 8.86		26.38 ± 12.34		79.50 ± 32.09	
Retired	16.29 ± 11.15		19.00 ± 12.23		15.29 ± 13.89		50.57 ± 34.18	
Employed/working	22.17 ± 12.98		22.17 ± 11.92		19.50 ± 14.27		63.83 ± 35.10	
P-value	0.504		0.198		0.295		0.275	
Other clinical characteristic								
Exposed to radiation								
Yes	21.50 ± 13.50		25.40 ± 12.06		20.00 ± 15.74		52.00 ± 30.75	
No	20.18 ± 11.83		122.45 ± 11.08		21.36 ± 12.13		36.73 ± 27.00	
P-value	0.814		0.567		0.825		0.852	
Radiation therapy								
Yes	18.20 ± 11.11		22.50 ± 12.43		18.10 ± 14.79		58.80 ± 35.50	
No	23.18 ± 13.45		25.09 ± 10.75		23.09 ± 12.70		58.80 ± 35.50	
P-value	0.369		0.615		0.416		0.416	
Multiple session radiation therapy								
Yes	23.44 ± 12.20		20.55 ± 13.54		20.44 ± 14.23		64.44 ± 37.47	
No	18.83 ± 12.61		26.33 ± 9.26		20.91 ± 13.78		66.08 ± 33.40	
P-value	0.411		0.259		0.94		0.917	

## Discussion

Total laryngectomy is associated with some important consequences. Disease-free survival and post-surgery quality of life are the prime concerns of the patients. However, vocal and swallowing rehabilitation has long been a major challenge in these patients, and it has become as important as cure and survival. In this study, the voice and dysphagia psychometric properties of the patients who had undergone total laryngectomy were evaluated using the VHI and DHI. Focusing on the patient’s experience of having a voice and swallowing problem, combined with their medical diagnosis, provides a broad, meaningful picture of the health of an individual and can assist healthcare workers in the decision-making process of care.

The VHI can be used to measure the therapeutic outcomes of voice therapy, as well as the severity of the voice problems. The VHI provides information on patients’ perception of either their voice quality changing after laryngectomy or the impact on their functional speech abilities. Clinical experience suggests that patients accept and adjust to these changes, and investigating patient perspectives may be helpful in more successful rehabilitation. In our study, we evaluated the internal consistency of the VHI tool, and the findings were inconsistent with the research that has reported well-to-excellent internal consistency for this index [11,12].

Lorenz et al. found higher VHI scores (up to 64.1 ± 9.6) with reflux severity. Later, Agarwal et al. discussed the role of treatment modalities including the surgical procedure and details such as neck dissection and pharyngeal reconstruction as well as radiation therapy on quality of life measured by the VHI and voice-related quality of life (V-RQoL) questionnaires. Although the surgery did not show significant results, postoperative radiotherapy was initially associated with a higher level of voice handicap. However, the same score during follow-up was significantly decreased due to reduced tissue flexibility in the early post-radiotherapy period. A further assessment by Agarwal et al. found long-term outcomes of the V-RQoL reported higher patient satisfaction with the crucial contribution of social support (about 80% V-RQoL excellent score and >75% minimal VHI score). The quality-of-life questionnaire discovered significantly better outcomes in both socio-emotional and functional domains in the tracheoesophageal patient group compared to the esophageal group (p = 0.01; p = 0.01, respectively) in post-laryngectomy rehabilitation [13].

Antin et al. [14] reported a low VHI total and a three-domain score than in our study (the total VHI score was 51 ± 28: 19 ± 10 functional, 16 ± 9 physical, and 16 ± 11 emotional). The mean VHI (10 scores) of patients who underwent total laryngectomy was greater than those who underwent chemoradiotherapy, radiotherapy, or laser surgery [15]. In our study, the VHI mean scores were in the severe range of voice handicap. The majority of included patients belonged to the severe group of VHI. However, there was a slight reduction in the emotional domain scores for the patients. Some studies contrast with our results and reported that most of the patients had a minimal handicap (VHI mean score: 24.65 ± 18.11) and fewer patients had severe voice handicap problems after total laryngectomy and voice restoration [16]. These findings suggest that voice restoration after laryngectomy can improve voice handicapping as well as the quality of life. Demographic factors such as age and gender of the patient did not have a significant effect on VHI domains, consistent with earlier findings [16,17]. Previous studies reported that newly laryngectomee patients reported a more severe voice handicap than those who had been operated on a long time ago [1,2]. Time since surgery is a reported confounder that was not analyzed in our study. Many factors affect the patient-perceived voice output after laryngectomy. Treatment modalities such as chemoradiotherapy, total laryngectomy, or laser surgery reduction; post-treatment factors such as treatment completion; and other factors such as voice sound, social and cultural factors, occupation, smoking habit, age factor, and post-treatment voice therapy gastroesophageal reflux may all influence patients’ perceptions of their voice quality [15,18].

The DHI assesses the psychosocial impact of oropharyngeal dysphagia. The discrimination of the severity of oropharyngeal dysphagia makes it a good tool to be used as a measure of intervention outcomes. In our study, the internal consistency of the DHI was excellent with possible item redundancy. Items representing different dysphagia-related aspects of daily life and referring to either the psychosocial aspect of dysphagia, such as emotional, functional, or physical aspects, were related to each other. According to several [19-21] investigations, DHI is the most reliable tool for assessing the psychosocial impact of dysphagic patients, and it is most suitable for clinical purposes given the 25 items and the use of a uniform simple scoring format. The Cronbach’s alpha of the DHI total score supported the assumption that DHI is a unidimensional measure.

A quality-of-life study [22] found that 46% of the patients present with moderate or severe dysphagia after total laryngectomy, and it was lower than voice problems (57%) found in patients. In our study, the majority of the patients were in the moderate severity category and had a higher mean DHI score. In our study, there was a marked difference in mean DHI scores between the different severity groups of patients, and the functional domain and severity groups showed significant mean DHI differences.

A high incidence of dysphagia after total and pharyngolaryngectomy was reported by Queija et al. [23], and they concluded that severe dysphagia impacts the mental health of patients. Another study also explained that dysphagia has a negative effect on the quality of life of patients who underwent total laryngectomy [24]. Radiation therapy besides total laryngectomy is a known risk factor for the development and severity of dysphagia and is part of the treatment regimens for many total laryngectomy patients. In our study, the percentage of patients who underwent postoperative radiotherapy was low. This may be a factor for the low overall DHI score in the severe category. However, the mean psychosocial subscale scores were found to be higher in severe dysphagia patients. More than emotional and physical aspects included patients in our study significantly reported functional problems in DHI assessment in the severity group. In our study, DHI scores were not significantly associated with clinical and demographic factors which agreed with the Persian-DHI study [20].

Among the limitations of this study is the sample size. Moreover, not including the possible confounding factors such as time since surgery did not allow generalizations of the findings. Large prospective observational studies with the inclusion of preoperative and postoperative risk factors and the use of multivariable analysis methods are warranted.

## Conclusions

In general, the psychosocial domains of the VHI and DHI are related to each other. Cronbach’s alpha showed that both the DHI and VHI had overall excellent reliability. Patients after laryngectomy are associated with higher mean DHI and VHI overall and functional subscale scores. The results showed that the DHI and VHI are reliable tools to assess the effects of dysphagia and voice problems on quality of life. However, this study failed to establish any relationship between clinical and demographic characteristics of the patients with DHI and VHI.
